# Transcriptional Characterization of Porcine *Leptin* and *Leptin Receptor* Genes

**DOI:** 10.1371/journal.pone.0066398

**Published:** 2013-06-18

**Authors:** Dafne Pérez-Montarelo, Almudena Fernández, Carmen Barragán, Jose L. Noguera, Josep M. Folch, M. Carmen Rodríguez, Cristina Óvilo, Luis Silió, Ana I. Fernández

**Affiliations:** 1 Departamento de Mejora Genética Animal, Instituto Nacional de Investigación y Tecnología Agraria y Alimentaria, Madrid, Spain; 2 Genètica i Millora Animal, Institut de Recerca i Tecnologies Agroalimentaries, Lleida, Spain; 3 Departament de Ciència Animal i dels Aliments, Facultat de Veterinària, Universitat Autònoma de Barcelona, Bellaterra, Spain; 4 Genètica Animal, Centre de Recerca en Agrigenòmica, Bellaterra, Spain; University of Cordoba, Spain

## Abstract

The leptin (LEP) and its receptor (LEPR) regulate food intake and energy balance through hypothalamic signaling. However, the LEP-LEPR axis seems to be more complex and its expression regulation has not been well described. In pigs, LEP and LEPR genes have been widely studied due to their relevance. Previous studies reported significant effects of SNPs located in both genes on growth and fatness traits. The aim of this study was to determine the expression profiles of LEP and LEPR across hypothalamic, adipose, hepatic and muscle tissues in Iberian x Landrace backcrossed pigs and to analyze the effects of gene variants on transcript abundance. To our knowledge, non porcine LEPR isoforms have been described rather than LEPRb. A short porcine LEPR isoform (LEPRa), that encodes a protein lacking the intracellular residues responsible of signal transduction, has been identified for the first time. The LEPRb isoform was only quantifiable in hypothalamus while LEPRa appeared widely expressed across tissues, but at higher levels in liver, suggesting that both isoforms would develop different roles. The unique LEP transcript showed expression in backfat and muscle. The effects of gene variants on transcript expression revealed interesting results. The LEPRc.1987C>T polymorphism showed opposite effects on LEPRb and LEPRa hypothalamic expression. In addition, one out of the 16 polymorphisms identified in the LEPR promoter region revealed high differential expression in hepatic LEPRa. These results suggest a LEPR isoform-specific regulation at tissue level. Conversely, non-differential expression of LEP conditional on the analyzed polymorphisms could be detected, indicating that its regulation is likely affected by other mechanisms rather than gene sequence variants. The present study has allowed a transcriptional characterization of LEP and LEPR isoforms on a range of tissues. Their expression patterns seem to indicate that both molecules develop peripheral roles apart from their known hypothalamic signal transduction function.

## Introduction

The leptin hormone, coded by the *LEP* gene, regulates energy balance, food intake and body weight [1]. Leptin is mainly secreted by white adipocytes into the blood stream. At hypothalamic level, it interacts with its receptor *LEPR* that encodes a signal transductor that activates the Janus kinases (JAK) and signal transducers and activators of transcription (STAT) as a response to leptin. Overall, these processes result in an increase of energy expenditure and physical activity and a reduction of the food intake, driving an adipose mass reduction [2]. However the role of the LEP-LEPR axis has been shown to be more complex. There are evidences revealing that leptin may act peripherally as well [3]. Although a single *LEP* isoform has been described in most mammals, six different *LEPR* isoforms have been identified in several species. Both, in human and mouse, the longer *LEPRb* isoform is mainly expressed in hypothalamus [4], [5] and appears to be the dominant signaling isoform regulating food intake and body weight [6], [7], [8], [9]. In human, expression of the short *LEPRa* isoform has been detected in most tissues; supposedly involved in leptin transport through the blood-brain barrier or in leptin degradation [10]. Indeed, it has been shown that in many tissues short *LEPR* isoforms predominate [11]. Nevertheless, the specific function and tissue distribution of short *LEPR* isoforms remain unclear in all species.

In pigs, *LEP* and *LEPR* genes have been widely studied due to their relevance on important economic traits such as growth and fatness [12], [13]. Our previous studies on an Iberian x Landrace experimental cross reported significant effects of SNPs located in both genes on pig productive traits [14], [15], [16]. A highly significant and strong additive effect on fatness and growth has been reported for *LEPR*c.1987C>T polymorphism in this population [14]. Moreover, the effect of this SNP on growth and fatness has been confirmed in very different genetic backgrounds (crossbred Iberian x Meishan, Duroc x Iberian and Duroc x Landrace/Large White pigs) and growth stages [17]–[20]. In addition, differential *LEPRb* expression according to this SNP was found in hypothalamus [15]. In the same Iberian x Landrace experimental intercross, effects of *LEP*g.1387C>T and joint effects of *LEP*g.1387C>T and *LEPR*c.1987C>T polymorphisms were detected on growth, fatness, body composition and fatty acid composition [16]. Despite their relevant role, the expression patterns of porcine *LEP* and *LEPR* isoforms across tissues as well as gene expression regulation have not been characterized so far.

The objective of the present study was to improve the characterization of porcine *LEP* and *LEPR* genes in order to better understand their potential biological roles. A search of short *LEPR* isoforms has been carried out and expression of *LEP* and *LEPR* isoforms has been characterized across five different tissues (backfat, liver, hypothalamus, *Longissimus dorsi* and diaphragm) in a backcross (BC) of Iberian x Landrace pigs. Previously, a set of reference genes has been tested in order to establish the most adequate control genes to evaluate *LEP* and *LEPR* gene expression differences across the five tissues. In addition, the variation of *LEP* and *LEPR* promoter regions and the differential expression of both genes conditional on several SNPs genotypes have also been investigated.

## Materials and Methods

### Ethics Statement

Animal manipulations were performed according to the Spanish Policy for Animal Protection RD1201/05, which meets the European Union Directive 86/609 about the protection of animals used in experimentation. Research protocols were approved by Animal Care and Use Committee of the Institut de Recerca i Tecnologies Agroalimentaries. The animals used in the present study grown in an experimental farm in good conditions and were fed according to their necessities. Electric stunning was used to ameliorate the suffering of the animals before sacrifice. The animals sacrifice took place at the PRIMAYOR slaughterhouse, property of the company “Primayor Foods S.L.” in Lleida, Spain. All the animals used and their information belong uniquely to the project AGL2011-29821-C02-02, and the slaughterhouse has no responsibility for them.

### Animal Material, DNA and RNA Extraction and cDNA Synthesis

Animals used in this study belong to a BC generated from the IBMAP population [21]. In brief, three Iberian boars were mated to 30 Landrace sows (F0) to produce 70 F1 animals. The BC was generated by mating 5 F1 boars with 25 Landrace sows to produce 187 BC animals. A total of 40 BC males belonging to the same batch were selected to identify new isoforms and to measure *LEP* and *LEPR* genes expression. Genomic DNA from parental pigs and the 40 selected BC animals was extracted from blood samples with a standard phenol: chloroform protocol, and used for promoter sequencing and polymorphisms genotyping. Samples of liver, hypothalamus, *Longissimus dorsi*, diaphragm and backfat (taken at the level of the fourth rib) from the BC animals were collected at slaughter at an average age of 179.9±2.6 days and 98.46±14.32 kg, immediately frozen in liquid nitrogen and stored at −80°C until analyzed. Total RNA was extracted from the five tissues using the RiboPure kit (Ambion, Austin, TX, USA) following the manufacturer’s recommendations. The total RNA was quantified using NanoDrop-100 spectrophotometer (NanoDrop Technologies, Wilmington, DE, USA). The integrity of the RNA was assessed using an Agilent 2100 Bioanalyzer device (Agilent technologies, Santa Clara, CA, USA). The cDNA synthesis was performed using the Superscript II enzyme (Invitrogen, Carlsbad, CA, USA) with random hexamers in a total volume of 20 µl containing 1.5 µg of total RNA, following the supplier’s instructions.

### Identification of Novel Porcine *LEPR* Isoforms

**Figure 1:**
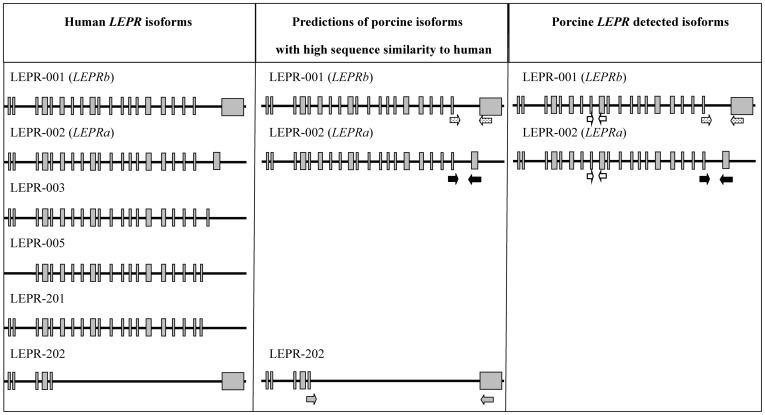
Schematic representation of the *LEPR* isoforms. Human *LEPR* isoforms, predictions of porcine *LEPR* isoforms showing high sequence similarity to human and detected porcine isoforms. The arrows represent the primers location for isoforms identification on the predicted porcine isoforms and for isoforms quantification on the porcine detected isoforms. Empty arrows represent *LEPRglobal* primers, arrows filled with black dots represent *LEPRb* primers, black filled arrows *LEPRa* primers and grey filled arrows primers designed to detect LEPR-202 isoform.

A total of six different *LEPR* isoforms have been detected in mouse and human. However, only the longest one (*LEPRb*) has been described in pig so far [1]. The identification of short *LEPR* isoforms conducted in the present study was based on the homology between human *LEPR* isoforms (GenBank: NG_015831.2) and porcine (GenBank: FN677933.1) *LEPR* gene sequence. Primer pairs were designed on highly conserved regions between human and pig (Table S1) for those isoforms containing alternative exons. The primer pair named LEPR19-20′ was designed to amplify a putative porcine short *LEPR* isoform, homologous to human short isoform *LEPR*a (also known as LEPR-002 at Ensembl). This short isoform is identical to the *LEPRb* except for a shorter alternative exon 20 (named exon 20′) (Figure 1). Another primer pair, called LEPR5-20 was designed to amplify a shorter isoform homologous to the human LEPR-202 isoform (Ensembl). In human, *LEPR*-202 isoform is identical to *LEPRb* from exons 1 to 5 and 20 but lacking the segment covering exons 6 to 19 (Figure 1). PCRs were carried out, in four samples of each analyzed tissue, in a 25 µl final volume containing 2.5 µl of cDNA, 1 unit of Taq polymerase (Biotools), specific buffer, 2.5 mM of dNTPs and 0.5 µM of each primer. The specific annealing temperature of each primer pair is shown in Table S1.

The ExPASy tool [22] was used to translate the mRNA sequence to protein and SMART tool [23] to predict differences in the protein domains between the isoforms.

### Promoters Sequence Analyses and Polymorphisms Identification

Genomic DNA from the parental animals of the IBMAP population, three Iberian boars and 30 Landrace sows, was used for sequencing the *LEP* and *LEPR* promoter regions. The Promoter Scan tool, PROSCAN version 1.7 [24], and Promoter Inspector software [25], were used to validate putative promoter regions in the 5′UTR of *LEP* and *LEPR* genes, in order to confirm the previously described location of the promoters of both genes. Two primer pairs, LEPpro1 and LEPpro2 (Table S1), were designed to amplify 900 bp, in two overlapped fragments, of the 5′ *LEP* region in the promoter described by Stachowiak et al. [26] according to the available sequence GenBank AF492499.2. *LEPR* promoter region sequencing was conducted in accordance with the pig *LEPR* promoter described by Lee et al [27]. According to the available *LEPR* gene sequence (GenBank: FN677933.1), three primer pairs (Table S1) were designed to amplify 1,266 bp, in three overlapped fragments, of the 5′ region of this gene. PCRs were carried out in a 25 µl final volume containing 100 ng of DNA, 1 unit of polymerase (Biotools) or HotStart polymerase (Quiagen), specific buffer, 2 mM of dNTPs and 0.5 µM of each primer. The specific annealing temperature of each primer pair is shown in Table S1. The PCR reactions were carried out in a GeneAmp PCR System 9700 (Applied Biosystems, Warrington, UK). The PCR products from the *LEP* gene were purified using the ExoSAP-IT® method (Affymetrix) and the *LEPR* promoter PCR products with the GFX™ PCR DNA purification kit (GE Healthcare, UK) according to the manufacturers’ protocol. All products were sequenced with both forward and reverse primers using the 3100 BigDye® Terminator v3.1 Matrix Standard in a 3730 DNA Analyzer (Applied Biosystems Warrington, UK). The obtained sequences were edited and aligned using the EditSeq and MegAlign packages of the WinStar software for the identification of polymorphisms.

Transcription factors (TF) binding sites were examined in both promoter regions using the Molecular Informatics Resource for the Analysis of Gene Expression website of the Institute for Transcriptional Informatics (IFTI Mirage website) [28]. The search was made with the IFTI Tfsite option and mammalian sites as query parameters.

### Gene Expression Quantification

Relative transcript quantification of samples from liver, *Longissimus dorsi*, diaphragm, hypothalamus and backfat samples of the 40 selected males was performed in 384 plates using the LightCycler®480 Real-Time PCR System (Roche Diagnostic, Mannheim, Germany). Real-time qPCR reactions were performed in a total volume of 20 µl containing 2.5 µl of cDNA (1/10 dilution), 10 µl of Roche LightCycler mix and a specific amount of primer pairs (0.3 µl for reference genes, 0.4 µl for *LEPRglobal* and 0.6 µl for *LEPRb*, *LEPRa* and *LEP* genes, at 5 µM dilution in all cases). All primer pairs used are detailed in Table S1. Standard PCR on cDNA were carried out to verify amplicon sizes. A non-template control, without cDNA, was included as negative control. Cycling conditions were 95°C for 10 min, followed by 45 cycles of 95°C (15 sec) and 60°C (1 min), when the fluorescence was acquired. Finally, a dissociation curve to test PCR specificity was generated by one cycle at 95°C (15 sec), followed by 60°C (20 sec) and ramped up to 95°C with the fluorescence acquired during the increase to 0.01°C/sec. Data were analyzed with LightCycler 480 software (Roche) using the second derivative method [29]. All points and samples were run in triplicates as technical replicates and dissociation curves were analyzed for each individual replicate. Single peaks in the dissociation curves confirmed the specific amplification of the primer pairs and the absence of primer dimers. PCR efficiency was estimated by standard curve calculation using four points of cDNA serial dilutions (1∶2, 1∶4 and 1∶8) of a pool of five samples (one from each tissue).

### Reference Genes Selection

A set of six genes *(GADPH, TBP, TOP2B, B2M, ACTB* and *eEF2*) commonly used as reference genes in porcine expression studies were selected from the literature [30], [31]. These genes are involved in different biological processes and functions and were selected to avoid genes belonging to the same pathways that may be co-regulated, because the geNorm algorithm used assumes no co-regulation of housekeeping genes. Their stability was evaluated across the five analyzed tissues. The primer pairs used were described in Kuijk et al. [32] and Erkens et al. [33], except for *eEF2* gene for which primers were designed according to AK240374 sequence (Table S1). A total of ten samples, two from each tissue, were used to measure the stability of the reference genes across tissues. The gene stability measures (M) were calculated using geNorm algorithm [34]. Those genes with the lowest M values have the most stable expression in each particular condition.

### *LEP* and *LEPR* Gene Expression Quantification

The relative expression measures of *LEP* and *LEPR* transcripts were determined in the five tissues for the 40 selected backcrossed pigs. Primers for *LEP* mRNA quantification were designed according to the available sequence (GenBank NM_213840.1) covering exons 2 and 3 (Table S1). Two different isoforms of the *LEPR* gene were analyzed: the *LEPRb* and the shorter one described here for the first time (*LEPRa*). Primer pairs were designed from the GenBank AF092422.1 porcine sequence between exons 18 and 20 for the *LEPRb*, and between exons 19 and 20′ for the *LEPRa* (Table S1). Moreover, the total *LEPR* expression (*LEPRglobal*) was measured by a primer pair designed between exons 8 and 9 which are shared by all isoforms detected so far (Table S1).

### Genotyping of Polymorphisms

The previously reported *LEPR*c.1987C>T and *LEP*g.1387C>T polymorphisms and the polymorphisms detected in the present study in *LEP* and *LEPR* promoter regions were genotyped in the 40 BC animals selected for the gene expression analyses. The *LEPR*c.1987C>T and *LEP*g.1387C>T polymorphisms genotypes were determined on DNA samples by pyrosequencing technology following the protocols described in Óvilo et al. [14] and Pérez-Montarelo et al. [16], respectively. The genotyping of the polymorphisms detected in *LEP* and *LEPR* promoter regions was conducted by sequencing, using the same primer pairs and conditions used for polymorphisms identification. Additionally, 11 females from the same BC were genotyped and included in the differential *LEPR* gene expression analysis conditional on *LEPR*g.35856G>A genotype. The Haploview software was used to estimate the linkage disequilibrium between the identified SNPs per gene [35].

### Differential Expression Analyses

Statistical analysis of gene expression was carried out following the method proposed by Steibel et al [36], which consists of the analysis of cycles to threshold values (*C_p_*), for the targets and housekeeping genes using a linear mixed model. The following model was used for analyzing the joint expression of the target (*LEP* and *LEPR*) and control (*ACTB* and *B2M*) genes in different tissues:
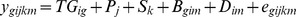
where 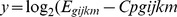), E is the efficiency of the PCR of each gene, *Cp* is the mean value obtained from the thermocycler software from the three replicates of *g*th gene in the *k*th plate in a sample collected from the *i*th tissue of the *m*th animal, *P_j_* and *S_k_* are the systematic effects of *j*th plate and *k*th gender (only for the differential *LEPR* expression analysis conditional on *LEPR*g.35856G>A genotype, where 11 females were included), *TG_ig_* is the specific effect of tissue *i* on the expression of gene *g*, *B_gim_* is a gene-specific random effect of the *m*th pig on the *i*th tissue, *D_im_* is a random tissue sample-specific effect common to all the genes, and *e_gijkm_* is a residual effect. A similar model was used for estimating the expression rates in each tissue of different genotypes of *LEP* and *LEPR* genes replacing *TG_ig_* by *TG_hig_*, which is the specific effect of genotype *h* on the expression of gene g in tissue *i*. Homogenous residual variances were assumed in these models according to the results of preliminary analyses with models fitting heterogeneous variances. Heteroscedasticity was discarded because of the small estimated differences among residual gene-specific variances and their marginal effects on the differential expression tests.

To test differences in the expression rate of genes of interest (*diff_TG_*) between classes (alternative tissues or genotypes) normalized by the housekeeping genes (*HK*), different contrasts were performed between the respective estimates of *TG* levels. Significance of *diff_TG_* estimates was determined with the *t* statistic. To obtain fold change values from de estimated *diff_TG_* values, the following equation was applied: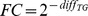. Asymmetric 95% confidence intervals (CI) were calculated for each *FC* value by using the standard error (SE) of the estimated difference: 95% CI from 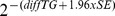 to 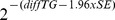.

## Results

### Identification of a Novel Porcine *LEPR* Isoform (*LEPRa*)

In order to identify novel porcine short *LEPR* isoforms two different primer pairs were designed: *LEPR*19-20′ and *LEPR*5-20. The shorter isoform homologous to human *LEPR*-202 could not be amplified (*LEPR*5-20 primer pair). However, a novel porcine *LEPR* isoform (*LEPRa),* homologous to human and murine *LEPRa,* was identified with LEPR19-20′ primer pair (Figure 1). This isoform is identical to the *LEPRb* described in pig from exons 1 to 19, but contains a different exon 20, probably shorter than the exon 20 of the *LEPRb,* according to its human homologous *LEPRa*. The predicted protein coded by the *LEPRb* contains 1,165 aminoacids while the protein coded by the *LEPRa* contains only 897. Even though the *LEPRb* is longer, the SMART predictions indicated that there were no differences in the domains present in both isoforms. According to this tool, both *LEPRb* and *LEPRa* have three FN3 (fibronectin type 3) domains and one transmembrane domain, all located in the region shared by both isoforms.

### *LEP* and *LEPR* Promoter Analyses

A total of 1,266 bp of the *LEPR* promoter region (positions 34669–35935 in the reference sequence GenBank: FN677933.1) were sequenced in the 40 BC animals used in the present study. This fragment contains the *LEPR* putative promoter, non-coding exons 1 and 2 and surrounding regions. The Promoter Scan and Promoter Inspector software confirmed the location of the predicted *LEPR* promoter within the analyzed region. A total of 16 polymorphisms were identified in this fragment (Table S2), 13 previously identified by Lee et al, [27] and three new ones (*LEPR*g.35782.indelGGAGGCCCCCGGGGCGA, *LEPR*g.35805A>G and *LEPR*g.35856). A total of 14 out of the 16 polymorphisms detected on the *LEPR* promoter were located within predicted TF binding sites (p<0.10) according to the IFTI Mirage website (Table S2).

A total of 900 bp of the *LEP* promoter region were also sequenced in the 40 animals. This fragment contains the *LEP* promoter, non-coding exon 1 and surrounding regions. The *LEP* promoter predicted by Promoter Scan agreed with the one reported by Stachowiak et al. [26] located within the analyzed region. A total of seven polymorphisms were identified in this region (Table S2). The search of potential transcription factors binding sites conducted with the IFTI Mirage website revealed that five of the seven detected polymorphisms were located within predicted TF binding sites (p<0.10) (Table S2).

### Gene Expression Quantification

#### Selection of the most suitable reference genes

In order to find the most stable reference genes to normalize gene expression measures across the five porcine tissues, the stability of six commonly used reference genes was tested: *GADPH, TBP, TOP2B, B2M, ACTB* and *eEF2.* The *eEF2* gene was discarded for further analyses due to amplifications problems. The stability (M values) provided by the geNorm software for the remaining five genes are represented in Figure S1. Three of the tested genes showed M values below 1.1 (*B2M, ACTB* and *TOP2B*). In order to choose the best pair of genes among those three most stable genes, their PCR efficiencies were taken into account (93%, 80% and 87% for *B2M, ACTB* and *TOP2B,* respectively). According to the M value and PCR efficiency, *B2M* and *ACTB*, were selected as the most suitable reference genes in our experiment, and were used for expression data normalization.

### Porcine *LEP* and *LEPR* Genes Expression Across Tissues

**Figure 2:**
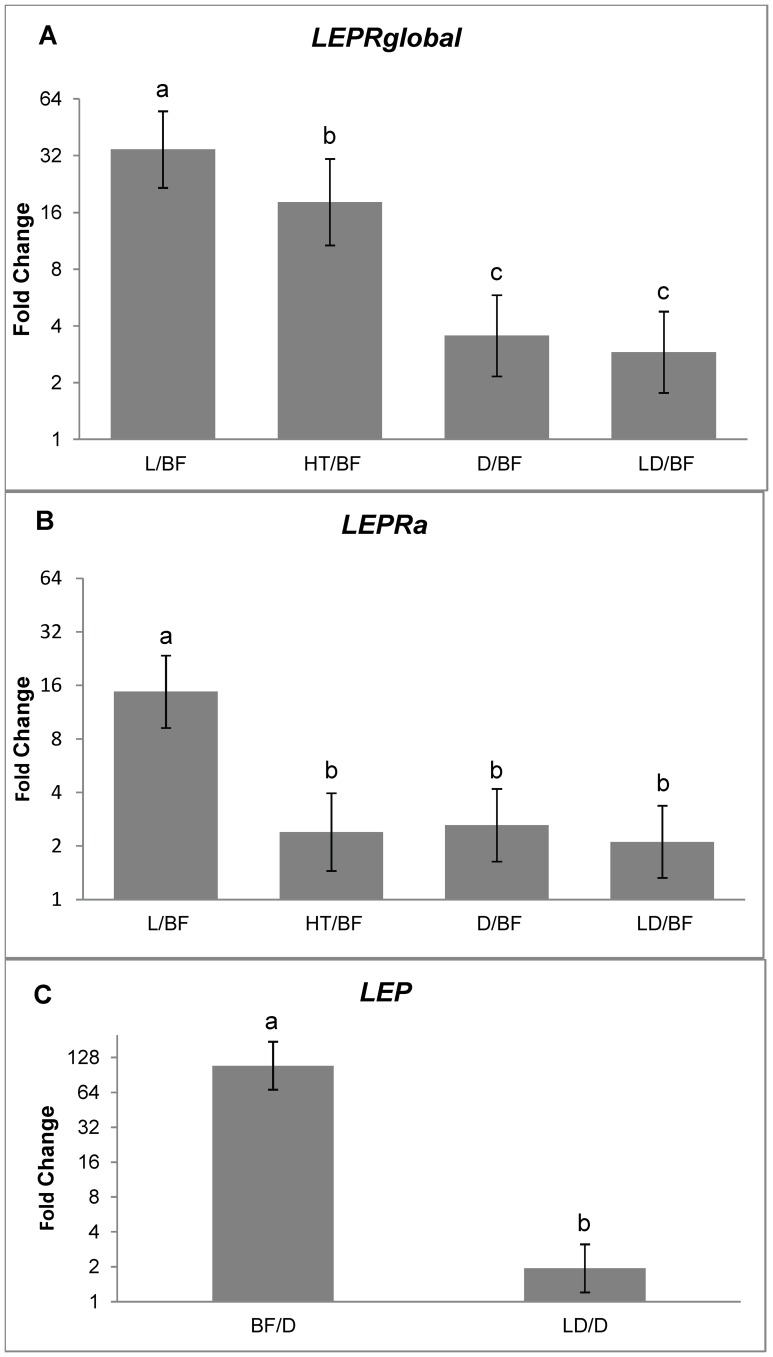
Pairwise comparison of gene expression values observed across the five tissues tested. **A)** *LEPRglobal* of all tissues related to backfat (the tissue that showed the lowest *LEPRglobal* expression). **B)** *LEPRa* of all tissues related to backfat (the tissue that showed the lowest *LEPRa* expression) **C)** *LEP* of all tissues related to diaphragm (the tissue that showed the lowest *LEP* expression). L: liver; BF: backfat; HT: hypothalamus; D: diaphragm; LD: *Longissimus dorsi*. Levels not connected by the same letter are significantly different (p<0.05).

Porcine *LEP* and *LEPR* genes expression levels showed a wide variation across the tested tissues (hypothalamus, backfat, liver, *Longissimus dorsi* and diaphragm). The results obtained for the global *LEPR* expression (*LEPRglobal*), that includes both detected isoforms (*LEPRa* and *LEPRb*), as well as, other isoforms that could potentially exist, are shown in Figure 2A (relative to the tissue that showed the lowest expression, backfat in this case) (Table S3). The highest *LEPRglobal* expression was found in liver followed by hypothalamus, muscles and finally backfat. Significant expression differences were detected among all tissues in the pairwise comparisons, except between both muscles (p-value = 0.37).

In addition, specific *LEPR* isoforms *(LEPRb* and *LEPRa)* expression differences were also measured. The *LEPRb* isoform showed very low expression levels, even undetectable, in backfat, liver, *Longissimus dorsi* and diaphragm and therefore could not be quantified in these tissues. This isoform could only be quantified in hypothalamus [15]. Conversely, *LEPRa* isoform expression could be detected in all the analyzed tissues. The significant differences of the *LEPRa* isoform expression across tissues are shown in Figure 2B in pairwise comparisons, relative to backfat, that showed the lowest *LEPRa* expression (Table S3). The highest *LEPRa* expression was detected in liver, followed by muscles, hypothalamus and backfat, which showed 15 times lower expression than liver. Significant differences were detected between liver and the remaining tissues (p<0.0001) and between backfat and the rest of the tissues (p<0.005).

The *LEP* expression analyses revealed large differences among tissues. This gene showed very low, almost undetectable, expression in hypothalamus and liver, therefore it could not be quantified in those tissues. Figure 2C represents the *LEP* unique isoform expression measures on backfat and *Longissimus dorsi* relative to diaphragm that showed the lowest expression (Table S3). LEP expression in backfat was more than 100 and 56 times higher than in diaphragm (p<0.0001) and *Longissimus dorsi,* respectively. Smaller, but still significant, were the differences detected between both muscles (p<0.05).

### Differential Expression Conditional on Genotypes

The expression differences of the different transcripts (*LEPRglobal, LEPRb*, *LEPRa,* and *LEP*) conditional on the genotypes of the previously analyzed polymorphisms, *LEPR*c.1987C>T and *LEP*g.1387C>T [14], [16], and the ones identified here in the promoter regions of both genes, were tested in the tissues where they showed quantifiable expression (Tables 1 and 2).

**Table 1:** Differential expression of *LEPR* transcripts conditional on *LEPRc.*1987C>T SNP genotypes.

Isoform	Tissue	Comparison	FC	Estimator	SE	95% CI	p-value
*LEPRglobal*	Backfat	CC-TT	5.447	−2.446	0.880	1.648–18.002	0.006
		CT-TT	2.984	−1.577	0.714	1.131–7.873	0.028
		CC-CT	1.826	−0.869	0.675	0.730–4.565	0.200
	Liver	CC-TT	7.783	−2.960	0.713	2.956–20.494	<.0001
		CT-TT	4.391	−2.135	0.664	1.782–10.819	0.002
		CC-CT	1.773	−0.826	0.496	0.903–3.478	0.098
*LEPRb*	Hypothalamus	CC-TT	2.715	−1.441	0.690	1.064–6.930	0.046
		CT-TT	2.794	−1.482	0.701	1.077–7.244	0.043
		CC-CT	0.970	0.049	0.605	0.430–2.210	0.946
*LEPRa*	Backfat	CC-TT	5.086	−2.347	0.819	1.673–15.464	0.005
		CT-TT	3.031	−1.600	0.669	1.222–7.518	0.018
		CC-CT	1.678	−0.747	0.629	0.714–3.943	0.237
	Hypothalamus	CC-TT	0.418	1.258	0.694	0.163–1.073	0.072
		CT-TT	0.313	1.678	0.708	0.119–0.818	0.019
		CC-CT	1.338	−0.420	0.611	0.583–3.070	0.493
	Liver	CC-TT	6.298	−2.655	0.775	2.197–18.061	0.001
		CT-TT	2.870	−1.521	0.671	1.153–7.144	0.025
		CC-CT	2.194	−1.134	0.575	1.005–4.791	0.050

FC: fold change; SE: standard error; CI: confidence interval.

**Table 2:** Differential expression of *LEPR* transcripts conditional on genotypes of different *LEPR* promoter SNPs.

SNP	Isoform	Tissue	Comparison	FC	Estimator	SE	95% CI	p-value
LEPR34996C>T	*LEPRglobal*	Backfat	CT-TT	0.356	1.488	0.503	0.180–0.706	0.003
		Liver	CT-TT	0.306	1.707	0.497	0.156–0.602	0.001
	*LEPRb*	Hypothalamus	CT-TT	0.418	−1.258	0.577	0.917–0.191	0.038
	*LEPRa*	Backfat	CT-TT	0.333	1.587	0.537	0.161–0.690	0.003
LEPR35856G>A	*LEPRglobal*	Liver	GA-GG	9.875	−3.304	0.456	5.316–18.343	<.0001
	*LEPRa*	Liver	GA-GG	15.770	−3.979	0.595	7.027–35.391	<.0001
LEPR35592G>A	*LEPRglobal*	Backfat	GA-AA	0.381	−1.391	0.455	0.708–0.205	0.003
		Liver	GA-AA	1.873	0.906	0.435	3.384–1.037	0.039
	*LEPRa*	Backfat	GA-AA	0.471	−1.087	0.497	0.925–0.239	0.030
		Liver	GA-AA	2.697	1.431	0.497	5.299–1.372	0.005
LEPR35657G>C	*LEPRa*	Liver	GC-CC	2.294	1.198	0.495	4.492–1.171	0.016

FC: fold change; SE: standard error; CI: confidence interval.

#### LEPR expression conditional on genotypes of LEPRc.1987C>T and LEPR promoter SNPs

**Figure 3:**
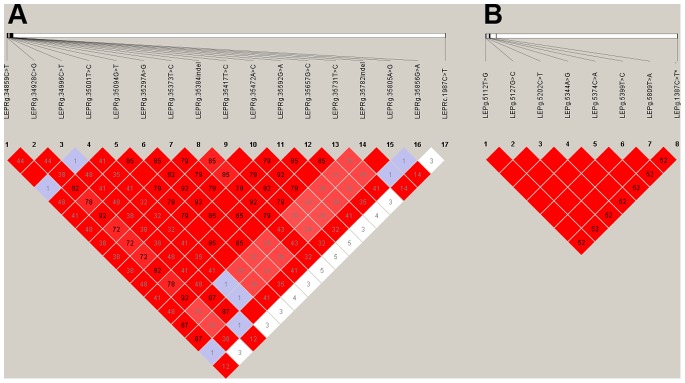
Linkage disequilibrium analysis among the SNPs detected for *LEPR* (A) and *LEP* (B) genes. The numbers in the boxes represent the linkage disequilibrium estimated with the r^2^ value. Those boxes without a number indicate a complete linkage.

The Haploview software showed that the *LEPR* promoter SNPs are highly linked (mean r^2^ = 0.6) (Figure 3A) and four groups of cosegregating SNPs could be identified (Table S2). Conversely, the *LEPR*c.1987C>T, located more than 65Kb far away from the promoter, and the promoter SNPs showed very low linkage (mean r^2^ = 0.06) (Figure 3A). Significant *LEPRglobal* differential expression was found in backfat (p<0.05) and liver (p<0.005) conditional on *LEPR*c.1987C>T genotype, where the C allele in both tissues increases the *LEPRglobal* gene expression (Table 1). The specific *LEPRb* expression conditional on *LEPR*c.1987C>T genotype in hypothalamus, was previously investigated in the same animal material, showing a higher *LEPRb* expression associated also to the C allele [15]. Similar results has been found in the present study (Table 1) using a more complex statistical model. Regarding *LEPRa* isoform, also significant differential expression was found in backfat (p<0.01) and liver (p<0.05) according to *LEPR*c.1987C>T genotype (Table 1). In both tissues, the C allele showed a higher expression than T, as shown in previous *LEPRglobal*. An effect of this SNP on *LEPRa* has also been detected in hypothalamus; however, the effects seem to differ from the one reported for the *LEPRb* isoform in hypothalamus. In hypothalamus, whereas the C allele is associated with a higher *LEPRb* expression, it results in a lower expression of *LEPRa,* suggesting a possible isoform specific regulation in this tissue. No significant differential expression was detected in muscle tissues for any isoform according to this SNP.

To test whether the polymorphisms identified in the promoter region of *LEPR* gene play a role in gene expression regulation, expression differences of *LEPRglobal, LEPRb* and *LEPRa,* conditional on these genotypes were also tested. The 16 polymorphisms identified in the *LEPR* promoter region were classified in four groups of cosegregating SNPs (Table S2) in the 40 backcrossed animals. The SNPs *LEPRg*.34996C>T, *LEPRg*.35592G>A, *LEPRg*.35657G>C and *LEPRg*.35856G>A were selected for differential expression analyses as representative of the four most informative groups of cosegregating polymorphisms (MAFs of 0.12, 0.22, 0.24 and 0.09, respectively). It is important to note that for all these promoter SNPs, one of the homozygous genotypes was detected in just one or none of the individuals; thereby these homozygous were discarded in further analyses.

Significant *LEPRglobal* expression differences were detected in liver and backfat conditional on *LEPRg*.34996C>T (p<0.05) and *LEPRg*.35592G>A (p<0.05) polymorphisms, however with opposite effects for the last SNP (Table 2). In addition, high *LEPRglobal* expression differences in liver were detected according to *LEPRg*.35856G>A, a SNP unidentified in previous studies. For this SNP, the GA genotype showed almost ten times higher expression than GG in liver. Regarding the specific *LEPR* isoforms, *LEPRb* resulted differentially expressed in hypothalamus according to *LEPR*g.34996C>T SNP, (Table 2), in the same sense that the observed for *LEPRglobal* in backfat and liver. Finally, significant *LEPRa* differential expression in liver and backfat were also detected for several polymorphisms. Expression differences of *LEPRa* in backfat tissue were found conditional on *LEPRg*.34996C>T and *LEPRg*.35592G>A SNPs, and in liver according to *LEPRg*.35856G>A, *LEPRg*.35592G>A and *LEPRg*.35657G>C (Table 2). As for *LEPRglobal*, the highest differences in hepatic gene expression were obtained for the *LEPRg*.35856G>A, where, same as before, the carriers of the GA genotype showed higher *LEPRa* expression than the homozygous GG. In order to validate this last result, due its large expression differences, eleven additional females were included into the analyses (6 AG and 5 GG animals). Note that only one animal of the whole pedigree was carrier of the AA genotype and therefore this genotype effect could not be tested. The addition of more samples allowed the validation of the expression differences detected revealing significant *LEPRa* differential expression (p<0.001) with a fold change of 15.77 between GA and GG genotypes (Table 2).

### *LEP* Expression According to *LEP*g.1387C>T and *LEP* Promoter SNPs

The results from the Haploview software showed that all SNPs located in the *LEP* promoter are completely linked (MAF = 0.30), conversely *LEP*g.1387C>T is not fully linked (mean *r* ^2^ = 0.5) (Figure 3B). No significant expression differences could be detected for *LEP* gene in the analyzed tissues, according to the previously identified *LEP*g.1387C>T SNP, and neither to the promoter polymorphisms (Tables S4 and S5).

## Discussion

The leptin hormone (*LEP*) interacts with its receptor (*LEPR*) to regulate food intake and energy expenditure influencing thereby important traits as growth and fatness [1]. Although *LEPR* gene presents several isoforms in other species, the *LEPRb* isoform is the best known and characterized. To our knowledge, none porcine *LEPR* isoform has been described rather than *LEPRb*. In the present study, a porcine short *LEPR* isoform has been described for the first time (*LEPRa*), homologous to human *LEPRa*. Compared with the porcine *LEPRb*, this novel isoform differs in the exon 20, in agreement with previous studies of *LEPR* isoforms in other species [4], [5]. The *LEPRa* and *LEPRb* isoforms identified in the present study share identical extracellular and transmembrane N-terminus regions, but differ in the intracellular C-terminus. The predicted FN3 and transmembrane domains are located in the shared region. However, the longer intracellular region of the *LEPRb* contains the tyrosine residues responsible for intracellular signal transduction at hypothalamic level. An isoform lacking these residues would not be able to transduce the *LEP* signal by the same mechanism than *LEPRb* [37].

To get further insights on the potential functions of these *LEPR* isoforms, their expression patterns across five different porcine tissues have been investigated. Previously, a selection of control genes for expression data normalization was conducted. The most critical step in measuring gene expression is an accurate normalization using suitable reference control genes. Even though numerous studies have evaluated different reference genes in several species, most of them have been directed towards specific types of tissues [30], [33]. The difficulty increases notably when searching for reference genes exhibiting constant RNA transcription across tissues [38], [39]. Even more when these tissues display great histological and metabolic differences, such as the five tissues analyzed. In the present study, the stability of five reference genes has been analyzed. According to the geNorm analyses and taking into account also PCR efficiencies of the five reference genes (*GADPH*, *TBP*, *TOP2B*, *B2M* and *ACTB*), *ACTB* and *B2M* genes were selected for normalizing the RT-PCR data.

The choice of a linear mixed model framework for estimating differential expression was justified by the complex design involving diverse experimental factors and biological and technical sources of variation. Simulation studies performed by Steibel et al. [36] showed that models fitting random sample effects (as *D_im_*) and random interaction between sample and gene factors (as *B_gim_*) provided better type I error rates and confidence intervals coverage than other alternative models of RT-PCR data analysis. The usefulness of this type of models in different experimental situations has been confirmed in recent studies [40], [41].

The *LEPRb* isoform was quantifiable only in hypothalamus, previously reported in the same animal material by Ovilo et al. [15]. Its expression was unquantifiable in liver, fat and muscle tissues. This result was expected as it is known that *LEPRb* isoform acts mainly on hypothalamic nuclei, and agrees with previous studies in other species [4], [5]. Even though, some authors reported the quantification of *LEPRb* expression in peripheral tissues in pigs [42], [43], they do not report isoform differences and it is likely that the expression measured corresponded to a mixture of isoforms, instead of specifically the long one [44], [45]. Moreover, this mixture of isoforms would mainly correspond to *LEPR* short isoforms, as it is known that *LEPRb* is much less abundant than the short forms in peripheral tissues [46].

Previous studies in other species reported that the short *LEPR* isoforms are widely expressed across all tissues [8], [44]. Our results support this idea and show a significantly higher expression level of the porcine *LEPRa* isoform in liver compared to the remaining tissues. Notably, this result contrasts with the results obtained for the *LEPRb* isoform. The fact that both isoforms have such a different expression pattern suggests that they may develop different roles on the LEP-LEPR axis.

Although liver is the tissue responsible for the handling and degradation of several hormones, it is not likely that the high expression of the *LEPRa* in the liver is related to leptin clearance. Several studies performed in human [47] and rodents [48], [49] concluded that the kidney is the main tissue of leptin clearance. Still, some authors have tried to find evidences of leptin degradation in other tissues like liver or spleen in human. Garibotto et al [50] supported the hypothesis that splanchnic organs (liver, spleen and small intestine) contribute in a significant way to leptin clearance when renal metabolic activity and function decline. In contrast, Jensen et al. [51] stated that even if small amounts of leptin are cleared by splanchnic organs in humans; they are minimal compared with kidney leptin clearance. A peripheral specific action of leptin in the liver could be a plausible explanation for this high *LEPRa* expression. Some studies proposed a role of leptin in liver cholesterol metabolism, downregulating cholesterol biosynthesis, upregulating cholesterol catabolism and decreasing plasma levels of very low-density lipoprotein [52]. *In vitro* and *in vivo* evidences proposed leptin peripheral roles in modulating liver lipoprotein receptor levels to ensure efficient lipid removal following a meal and contributing to the dynamics of lipid distribution and utilization [53]. Within the frame of these leptin peripheral roles, *LEPRa* would be necessary in the liver to detect and respond to different amounts of leptin triggering the specific downstream actions.

The performed *LEPRglobal* expression measures allowed the estimation of the relation among total amount of *LEPR* isoforms and the specific *LEPRa* and *LEPRb*. High or moderate correlations were detected between *LEPRa* and *LEPRglobal* expression across tissues except in the hypothalamus. Actually, the correlation between both measures was 0.87 and 0.90 in backfat and liver respectively (p<0.0001), and 0.4 in both muscles (p<0.05). The high correlation found in backfat and liver suggest that *LEPRa* is the predominant isoform in these tissues, or if there are other short isoforms, they would show low expression or are regulated in the same way. The results found in muscles suggest that there are other isoforms, apart from *LEPRa* expressing in both muscles. As mentioned before, no significant correlation was found between *LEPRa* and *LEPRglobal* in hypothalamus (0.321; p>0.1), but it should be taken into account that *LEPRb* is almost exclusively expressed in this tissue and included in the *LEPRglobal* measure, but not in *LEPRa* measure. Even more, *LEPRa* and *LEPRb* isoforms measures do not appear significantly correlated (−0.27; p>0.1), suggesting an isoform specific regulation at the hypothalamic level. A plausible explanation of the results obtained could be an autorregulatory mechanism, as both isoforms seem to develop different functions in hypothalamus. Therefore, an increase of *LEPRb* in the hypothalamus would inhibit *LEPRa* expression in this tissue. These mechanisms of autoregulation among isoforms have been previously reported for genes such as *Pax6* [54] and *ZFHX1A* [*55*].

The only *LEP* transcript (expands from exon 2 to exon 3) showed a significant higher expression on backfat compared to the other tested tissues. This highly specific backfat *LEP* expression was expected as it is known that *LEP* is mainly synthesized by white adipocytes. However, the detection of *LEP* expression in both muscles, even at low levels compared to backfat, gives more evidence of the potential peripheral roles of this hormone.

In order to get other insights on *LEP* and *LEPR* transcriptional regulation, gene expression differences conditional on several polymorphisms have been investigated. Our previous study revealed that *LEPRb* hypothalamic expression is conditioned by *LEPR*c.1987C>T polymorphism, the animals carrying the T allele showed a lower expression than carriers of the C allele [15]. This result is confirmed in the present study using a more suitable statistical approach. A similar effect of this SNP was also found on *LEPRa* expression in the liver and backfat. Interestingly, an opposite effect of *LEPR*c.1987C>T on *LEPRa* hypothalamic expression was found, the TT carriers showed higher expression that CT. In agreement with these conflicting result, no effect of *LEPRc.*1987C>T was found when analyzing the *LEPRglobal* expression in hypothalamus. These results support the hypothesis of isoform specific regulation at the hypothalamic level. The *LEPR* promoter was previously described by Lee et al. [27], but differential expression analyses have never been performed before conditional on SNP genotypes located in this region. Here, 16 polymorphisms have been identified in the *LEPR* promoter region, 14 of which are located within potential TF binding sites. The analysis of these polymorphisms revealed several significant differential expressions in hypothalamus, liver and backfat. However, it should be taken into account that for all these SNPs only two segregating genotypes appeared (one of the homozygous was found in one or none individuals), and most of them are highly linked (Figure 3A). Additionally, multiple statistic tests have been performed, therefore some of these results could be significant by random and should be taken with caution. Nonetheless, a high consistency was found between *LEPRa* and *LEPRglobal* expression differences according to the promoter SNPs analyzed within tissue (liver and backfat). The results obtained for the novel *LEPR*g.35856G>A promoter SNP on both *LEPRa* and *LEPRglobal* in liver were especially relevant, due to the high expression differences detected. This SNP is representative of two SNPs, *LEPRg.*35856G>A and *LEPRg*.35001T>C, that are predicted to fall within TF binding sites (Table S2). More specifically, the *LEPR*g.35856G>A polymorphism falls within a predicted target for the *CREB* transcription factor (p<0.05; Table S2), which is implicated in expression regulation of several genes affecting appetite [56]. The results obtained for *LEPR* expression analyses strongly suggest a tissue and isoform specific regulation focused mainly on the effects of the exonic *LEPRc*.1987C>T SNP on the regulation of both *LEPRb* and *LEPRa* expression in different tissues, but with opposite effects on both isoforms in hypothalamus, and the promoter *LEPR*g.35856G>A SNP that would specifically regulate *LEPRa* expression in hepatic tissue.

Finally, non-differential expression of *LEP* conditional on any of the analyzed polymorphisms could be detected. These results agree with the study of Stachowiak et at. [26], which did not find association of the *LEP* expression levels in subcutaneous fat with the polymorphisms identified in the *LEP* promoter region. Although Liu et al. [57] reported differential expression of the *LEP* gene in subcutaneous fat tissue due to a polymorphism detected outside the functional promoter region; our results seem to indicate that gene expression regulation is likely affected by other mechanisms rather than gene sequence variants. Diet and changes in nutrient availability result in rapid alterations in *LEP* autoregulation mechanisms [58]. In addition, the adipocyte volume and several metabolites such as insulin, glucose, glucocorticoids or cytokines have been shown to affect *LEP* expression [59].

An overview of the results obtained in the present study suggests an implication of both molecules (LEP and LEPR) on liver metabolism. In addition, the specific expression patterns of the *LEPR* isoforms highlight the idea that they are involved in different biological pathways, supporting previous studies that suggest peripheral roles of porcine LEP and LEPR. In fact, previous studies reported effects of *LEP* and *LEPR* genes polymorphisms on fatty acid composition in backfat and muscle suggesting local effect of LEP-LEPR on fatty acids metabolism, specifically on oleic fatty acid level [16]. Similarly, Galve et al. [20] suggested a local effect of LEP and LEPR in skeletal muscle to explain the changes in the proportions of saturated and unsaturated fatty acids observed in different muscle tissues. The present study has allowed a transcriptional characterization of *LEP* and *LEPRa* and *LEPRb* isoforms on a range of tissues. Complementary functional studies would be required in order to determine the peripheral *LEP* and *LEPR* gene function and regulation.

## Supporting Information

Figure S1 **Average expression stability values of the reference genes tested according to the geNorm analysis.** Those genes with stability values lower than 1.5 could be considered as appropriate reference genes.
(TIF)

Table S1 **Primer pairs and PCR conditions for expression analyses,** ***LEPR*** **isoform detection and promoters sequencing.**
(DOC)

Table S2 **Description of the polymorphisms detected in the promoter regions of** ***LEP*** **and** ***LEPR*** **genes.**
(DOCX)

Table S3 **Pairwise comparison of gene expression values observed across the five tissues tested for** ***LEPRglobal, LEPRa*** **and** ***LEP*** **isoforms.**
(DOCX)

Table S4 **Differential** ***LEP*** **expression conditional on** ***LEPg.1387T>C*** **genotype.**
(DOCX)

Table S5 **Differential** ***LEP*** **expression conditional on** ***LEP*** **promoter SNPs.**
(DOCX)
