# Supplementary figures and images for: Transcriptional Characterization of Porcine Leptin and Leptin Receptor Genes
Source: PLoS One. 2013 Jun 18;8(6):e66398. doi: 10.1371/journal.pone.0066398 (PMC3688923; doi:10.1371/journal.pone.0066398)

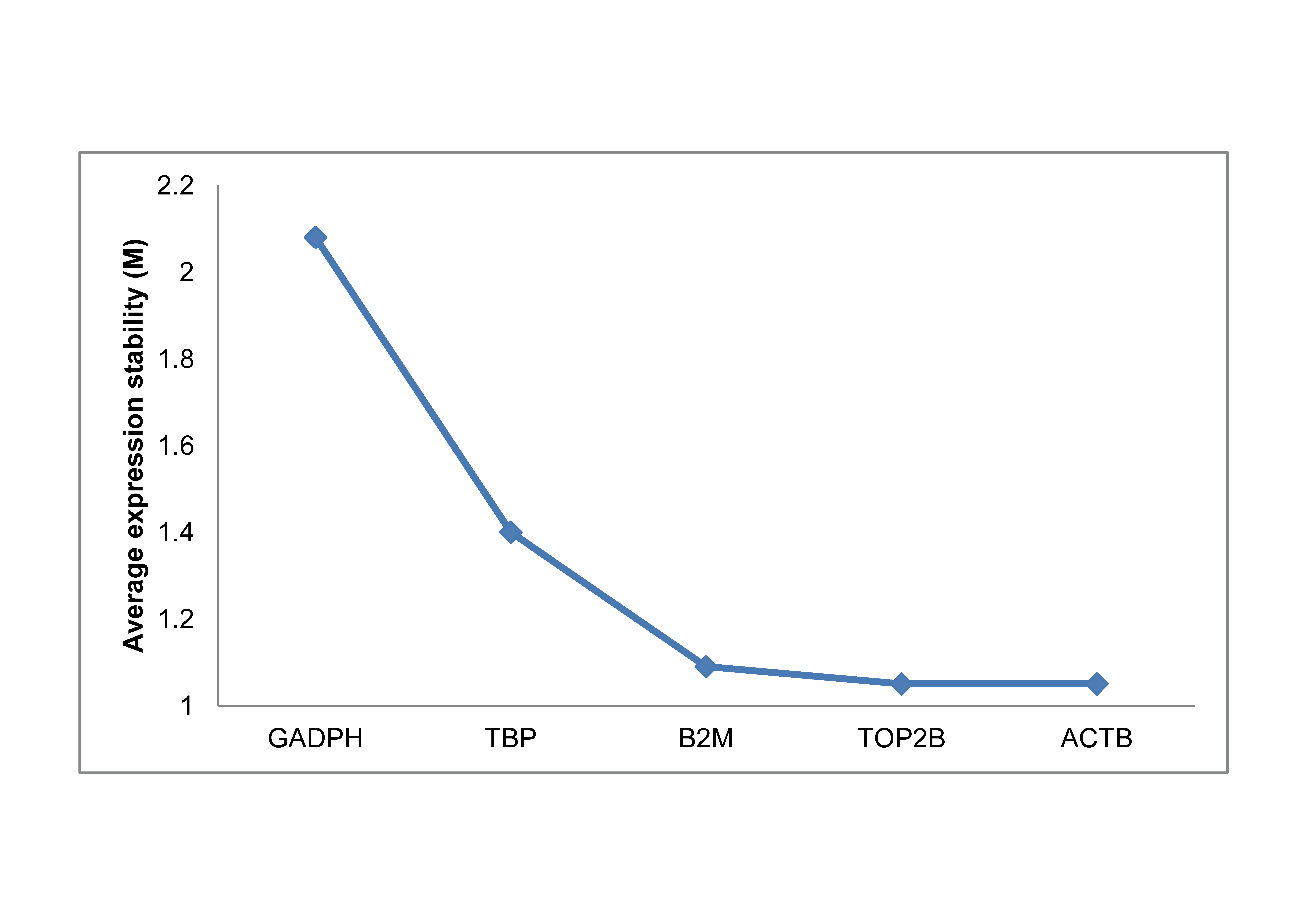

Supplement: Figure S1 — Average expression stability values of the reference genes tested according to the geNorm analysis. Those genes with stability values lower than 1.5 could be considered as appropriate reference genes. (TIF) [file pone.0066398.s001.tif]
