# Supplementary material for: Transcriptional Characterization of Porcine Leptin and Leptin Receptor Genes
Source: PLoS One. 2013 Jun 18;8(6):e66398. doi: 10.1371/journal.pone.0066398 (PMC3688923; doi:10.1371/journal.pone.0066398)
Supplement: Table S1 — Primer pairs and PCR conditions for expression analyses, LEPR isoform detection and promoters sequencing. (DOC) [file pone.0066398.s002.doc]

**Supplementary table S1.**

| Fragment | Primer sequence (5’ to 3’) | Size, bp | Ta,  ºC | Eff., % |
| --- | --- | --- | --- | --- |
| Reference genes | |  |  |  |
| *ACTB* | Fw:TCTGGCACCACACCTTCT Rv:GATCTGGGTCATCTTCTCAC | 114 | 60 | 80 |
| *B2M* | Fw:TTCACACCGCTCCAGTAG Rv:CCAGATACATAGCAGTTCAGG | 166 | 60 | 93 |
| *TOP2B* | Fw:AACTGGATGATGCTAATGATGCT Rv:TGGAAAAACTCCGTATCTGTCTC | 137 | 60 | 87 |
| *GADPH* | Fw:TCGGAGTGAACGGATTTG Rv:CCTGGAAGATGGTGATGG | 219 | 60 | 87 |
| *TBP* | Fw:GATGGACGTTCGGTTTAGG Rv:AGCAGCACAGTACGAGCAA | 124 | 60 | 90 |
| *eEF2* | Fw:CGACACTTTGAGACTGTCCAGACT Rv: AAGTGGGCCCCAGAAACC | 80 | 60 | - |
| Expression analyses | |  |  |  |
| *LEP* | Fw:GCTTTGGCCCTATCTGTCCTAC Rv:AAGTCCAAACCGGTGACCCT | 153 | 60 | 87 |
| *LEPRb* | Fw:GAAAAACACCGGAATGATGC Rv:AAAAGAAGAGGGCCAAATGTC | 239 | 60 | 81* |
| *LEPRa* | Fw:AAGCTATTTTGGGAAGATGTT Rv:ATGATGGCAAGTTGGTAGATT | 139 | 60 | 96 |
| *LEPRglobal* | Fw:GACTGGAGCACCCCCTTTACTT Rv:GCTAACATGGTCACCCACAACA | 207 | 60 | 98 |
| *LEPR* isoform detection | |  |  |  |
| LEPR*19-20’* | Fw:GGAGTGGGGAAACCGAAGATAA  Rv:CATGACCAGGCAAATGACAAAG | 363 | 56 |  |
| LEPR*5-20* | Fw:ATACAGTGCTGGATGAAAGAGGA Rv:CACTGCTGCGTTGGTCACTAATA | 324 | - |  |
| *LEP* promoter sequencing | |  |  |  |
| LEPpro1 | Fw:CTCATCCGCTCCGCACCATCACC Rv:GGGGCAAAGCTACCGCAATCCAGA | 681 | 62 |  |
| LEPpro2 | Fw:GGTGCGGCCCCCAAAAGATAAAT Rv:GGCGGCTGGCAGGGATGAGAAAT | 505 | 62 |  |
| LEPR promoter sequencing | |  |  |  |
| LEPRpro1 | Fw:TCTGCACACAGTAGGTCCTCA Rv:CGGTCCTTTACCCAGGTGTA | 514 | 58 |  |
| LEPRpro2 | Fw:CTGGGTGAAACATCCTTGGTGACA Rv:GAGCGGGAAAAGGGGGAAGTAAGG | 731 | (58-68) | |
| LEPRpro3 | Fw:CGCCGCCGCCATCTCTG Rv:GCATCCAAACCTACAAATCCTTCC | 436 | (58-68) | |

Ta: annealing temperature; Eff: Efficiency. (*) Efficiency in hypothalamus, the only tissue where *LEPRb* could be meassured.
