# Supplementary material for: Transcriptional Characterization of Porcine Leptin and Leptin Receptor Genes
Source: PLoS One. 2013 Jun 18;8(6):e66398. doi: 10.1371/journal.pone.0066398 (PMC3688923; doi:10.1371/journal.pone.0066398)
Supplement: Table S2 — Description of the polymorphisms detected in the promoter regions of LEP and LEPR genes. (DOCX) [file pone.0066398.s003.docx]

**Supplementary table S2.**

| Position | MAF | Predicted TF binding sites | Cosegregating group |
| --- | --- | --- | --- |
| *LEPR* promoter (Reference sequence FN677933.1) | | |  |
| 34859g.C>T | 0.121 | INSM1 (6.46e-01) | 1 |
| 34928g.C>G | 0.216 | NFAT-γ-INF (9.56e-03), NFATc3 (6.03e-01) and PEA3 (7.09e-01) | 2 |
| 34996g.C>T | 0.121 | SOX (7.08e-01) | 1 |
| 35001g.T>C | 0.094 | SOX (7.08e-01) | 3 |
| 35094g.G>T | 0.216 | - | 2 |
| 35297g.A>G | 0.243 | AP-2-α/ γ (9.96e-01) and MIZ-1a (4.60e-01) | 4 |
| 35373g.T>C | 0.216 | PEA3 (4.06e-01), MIZ-1c (4.60e-01), α -INF.2 (9.15e-01), Tst-1 consensus (7.08e-01), ε-globin (3.79e-02) | 2 |
| 35384g.indel CCCCTCCACTC | 0.243 | MAZ (3.42e-01), NeuroD1-fp4 (3.79e-02), SP1-GPC (2.04e-02), SP1-NPY (1.43e-01), p300-consensus (1.43e-01), AP-2 (4.59e-01), SP1-cyclin-D2 (2.04e-02) and H4TF-1-FVII (2.04e-02) | 4 |
| 35417g.T>C | 0.243 | CSRNP (4.60e-01) and Nkx-3.2 (9.75e-01) | 4 |
| 35472g.A>C | 0.243 | - | 4 |
| 35592g.G>A | 0.216 | γ -globin (4.60e-01) | 2 |
| 35657g.G>C | 0.243 | AP-2 (9.15e-01), GCF (9.15e-01), SP1-complement (1.43e-01) and 57bp-URS-heptam (2.65e-01) | 4 |
| 35731g.T>C | 0.216 | M-box (2.04e-02) and CSBP-2 (5.00e-01) | 2 |
| 35782g.indelGGAGGCCCCCGGGGCGA | 0.243 | AP2- α / γ (9.96e-01) and MED-1 (7.09e-01) | 4 |
| 35805g.A>G | 0.243 | LF-A1 (9.15e-01) and ELP/SF1/FTZ-F1 (7.40e-02) | 4 |
| 35856g.G>A | 0.094 | CREB –IL6 (5.54e-03) and E1A (7.09e-01) | 3 |
| *LEP* promoter (Reference sequence AF492499) | | |  |
| 5112g.T>G | 0.30 | - | 1 |
| 5127g.G>C | 0.30 | WT1 (6.27e-02), U-prosaposin (2.70e-02), MED-1 (5.83e-01) and AP-1-involucrin (1.03e-01) | 1 |
| 5202g.C>T | 0.30 | PEA3 (5.83e-01), Pu box (3.54e-01) and ESE-1 (6.67e-01) | 1 |
| 5344g.A>G | 0.30 | TBX5 (6.68e-01) | 1 |
| 5374g.C>A | 0.30 | INSM1 (4.59e-01) and AP2- α / γ (9.80e-01) | 1 |
| 5399g.T>C | 0.30 | - | 1 |
| 5809g.T>A | 0.30 | EBF/Olf.1 (1.03e-01) | 1 |

MAF: Minor allele frequency; TF: Transcription factor.
