# Supplementary material for: Transcriptional Characterization of Porcine Leptin and Leptin Receptor Genes
Source: PLoS One. 2013 Jun 18;8(6):e66398. doi: 10.1371/journal.pone.0066398 (PMC3688923; doi:10.1371/journal.pone.0066398)
Supplement: Table S3 — Pairwise comparison of gene expression values observed across the five tissues tested for LEPRglobal, LEPRa and LEP isoforms. (DOCX) [file pone.0066398.s004.docx]

**Supplementary table S3.**

| **Isoform** | **Comparison** | **FC** | **Estimator** | **SE** | **95% CI** | **p-value** |
| --- | --- | --- | --- | --- | --- | --- |
| *LEPRglobal* | L/BF | 34.5569 | -5.1109 | 0.3439 | 21.658-55.136 | <.0001 |
|  | L/LD | 11.9125 | -3.5744 | 0.3444 | 7.461 -19.019 | <.0001 |
|  | L/D | 9.7297 | -3.2824 | 0.3443 | 6.094 - 15.532 | <.0001 |
|  | L/HT | 1.9041 | -0.9291 | 0.3680 | 1.154 - 3.139 | 0.0123 |
|  | HT/BF | 18.1475 | -4.1817 | 0.3883 | 10.708 - 30.755 | <.0001 |
|  | HT/LD | 6.2563 | -2.6453 | 0.3888 | 3.689 - 10.610 | <.0001 |
|  | HT/D | 5.1099 | -2.3533 | 0.3887 | 3.013 - 8.664 | <.0001 |
|  | D/BF | 3.5517 | -1.8285 | 0.3658 | 2.160 - 5.838 | <.0001 |
|  | D/LD | 1.2243 | -0.2920 | 0.3663 | 0.744 - 2.013 | 0.4262 |
|  | LD/BF | 2.9007 | -1.5364 | 0.3659 | 1.760-4.768 | <.0001 |
| *LEPRa* | L/BF | 14.7629 | -3.8839 | 0.3442 | 9.248 - 23.564 | <.0001 |
|  | L/LD | 6.9900 | -2.8053 | 0.3421 | 4.391 - 11.125 | <.0001 |
|  | L/D | 5.6345 | -2.4943 | 0.3442 | 3.529 - 8.993 | <.0001 |
|  | L/HT | 6.1509 | -2.6208 | 0.3690 | 3.725 - 10.154 | <.0001 |
|  | HT/BF | 2.4001 | -1.2631 | 0.3710 | 1.449 - 3.973 | 0.0008 |
|  | HT/LD | 1.1364 | -0.1845 | 0.3690 | 0.688 - 1.876 | 0.6176 |
|  | HT/D | 0.9161 | 0.1265 | 0.3710 | 0.553 - 1.516 | 0.7336 |
|  | D/BF | 2.6201 | -1.3896 | 0.3463 | 1.636 - 4.194 | <.0001 |
|  | D/LD | 1.2406 | -0.3110 | 0.3442 | 0.777 - 1.980 | 0.3673 |
|  | LD/BF | 2.1120 | -1.0786 | 0.3442 | 1.323 - 3.371 | 0.0020 |
| *LEP* | BF/D | 109.0390 | -6.7687 | 0.3499 | 67.785 - 175.399 | <.0001 |
|  | BF/LD | 56.2546 | -5.8139 | 0.3501 | 34.961 - 90.515 | <.0001 |
|  | LD/D | 1.9382 | -0.9547 | 0.3502 | 1.204 - 3.119 | 0.0078 |

BF: backfat; D: diaphragm; LD: *Longissimus dorsi*; FC: fold change; SE: standard error; CI: confidence interval.
