# Supplementary material for: Transcriptional Characterization of Porcine Leptin and Leptin Receptor Genes
Source: PLoS One. 2013 Jun 18;8(6):e66398. doi: 10.1371/journal.pone.0066398 (PMC3688923; doi:10.1371/journal.pone.0066398)
Supplement: Table S4 — Differential LEP expression conditional on LEPg.1387T>C genotype. (DOCX) [file pone.0066398.s005.docx]

**Supplementary table S4.**

|  | FC | Estimator | SE | 95% CI | p-value |
| --- | --- | --- | --- | --- | --- |
| BF CC-TT | 1.2050 | -0.269 | 0.6813 | 0.4775-3.0406 | 0.6937 |
| BF TC-TT | 0.7363 | 0.4416 | 0.6264 | 0.3144-1.7245 | 0.4823 |
| BF CC-TC | 1.6365 | -0.7106 | 0.674 | 0.6550-4.0887 | 0.2939 |
| BF a | 1.0977 | -0.1345 | 0.3407 | 0.6910-1.7438 | 0.6937 |
| BF d | 0.6708 | 0.5761 | 0.5543 | 0.3159-1.4244 | 0.3008 |
| D CC-TT | 0.9829 | 0.02494 | 0.6954 | 0.3821-2.5281 | 0.9715 |
| D TC-TT | 0.6643 | 0.5902 | 0.6321 | 0.2814-1.5678 | 0.3524 |
| D CC-TC | 1.4797 | -0.5653 | 0.6649 | 0.5996-3.6515 | 0.397 |
| D a | 0.9914 | 0.01247 | 0.3477 | 0.6182-1.5900 | 0.9715 |
| D d | 0.6700 | 0.5777 | 0.5477 | 0.3184-1.4101 | 0.2936 |
| LD CC-TT | 0.8551 | 0.2258 | 0.6942 | 0.3330-2.1959 | 0.7456 |
| LD TC-TT | 0.5930 | 0.7539 | 0.6322 | 0.2512-1.3998 | 0.2355 |
| LD CC-TC | 1.4420 | -0.5281 | 0.6663 | 0.5832-3.5654 | 0.4296 |
| LD a | 0.9247 | 0.1129 | 0.3471 | 0.5771-1.4819 | 0.7456 |
| LD d | 0.6413 | 0.641 | 0.5489 | 0.3042-1.3518 | 0.2453 |

BF: backfat; D: diaphragm; LD: *Longissimus dorsi*; FC: fold change: SE: standard error; CI: confidence interval.
