# Supplementary material for: Transcriptional Characterization of Porcine Leptin and Leptin Receptor Genes
Source: PLoS One. 2013 Jun 18;8(6):e66398. doi: 10.1371/journal.pone.0066398 (PMC3688923; doi:10.1371/journal.pone.0066398)
Supplement: Table S5 — Differential LEP expression conditional on LEP promoter SNPs. (DOCX) [file pone.0066398.s006.docx]

**Supplementary table S5.**

|  | FC | Estimator | SE | 95% CI | Pr > \|t\| |
| --- | --- | --- | --- | --- | --- |
| BF GG-TT | 1.7564 | -0.8126 | 0.9317 | 0.4953-6.2278 | 0.3851 |
| BF TG-TT | 0.7970 | 0.3274 | 0.6158 | 0.3452-1.8398 | 0.596 |
| BF GG-TG | 2.2038 | -1.1400 | 0.986 | 0.5773-8.4126 | 0.2502 |
| BF a | 1.3253 | -0.4063 | 0.4659 | 0.7038-2.4957 | 0.3851 |
| BF d | 0.6014 | 0.7337 | 0.6772 | 0.2396-1.5090 | 0.2811 |
| D GG-TT | 1.7416 | -0.8004 | 0.936 | 0.4883-6.2116 | 0.3944 |
| D TG-TT | 1.2074 | -0.2719 | 0.6078 | 0.5287-2.7572 | 0.6555 |
| D GG-TG | 1.4423 | -0.5284 | 0.977 | 0.3825-5.4389 | 0.5897 |
| D a | 1.3197 | -0.4002 | 0.468 | 0.6988-2.4923 | 0.3944 |
| D d | 0.9149 | 0.1283 | 0.6655 | 0.3704-2.2596 | 0.8475 |
| LD GG-TT | 2.5448 | -1.3476 | 0.9358 | 0.7137-9.0739 | 0.1561 |
| LD TG-TT | 0.7228 | 0.4682 | 0.6081 | 0.3164-1.6513 | 0.4458 |
| LD GG-TG | 3.5258 | -1.8180 | 0.9774 | 0.9345-13.3029 | 0.0691 |
| LD a | 1.5952 | -0.6738 | 0.4679 | 0.8448-3.0123 | 0.1561 |
| LD d | 0.4541 | 1.1390 | 0.6661 | 0.1837-1.1224 | 0.0927 |

BF: backfat; D: diaphragm; LD: *Longissimus dorsi*; FC: fold change; SE: standard error; CI: confidence interval.
